# Unveiling the association between angiogenic imbalance in the gingival crevicular fluid in maternal periodontitis and spontaneous preterm birth

**DOI:** 10.3389/fdmed.2025.1625995

**Published:** 2025-07-29

**Authors:** Daniela Albers, María José Bendek, Marcela Hernández, Diego Prieto, Carolina Rojas, María Luisa Mizgier, Patricia Hernández, Sebastián E. Illanes, Alejandra Chaparro

**Affiliations:** ^1^ Department of Oral Pathology and Conservative Dentistry, Periodontics, Faculty of Dentistry, Universidad de Los Andes, Santiago, Chile; ^2^ Center for Biomedical Research and Innovation (CIIB), Universidad de Los Andes, Santiago, Chile; ^3^ Laboratory of Periodontal Biology and Department of Pathology and Oral Medicine, Faculty of Dentistry, Universidad de Chile, Santiago, Chile; ^4^ Department of Conservative Dentistry, Faculty of Dentistry, Universidad de Chile, Santiago, Chile; ^5^ Program in Biology of Reproduction, Faculty of Medicine, Universidad de Los Andes, Santiago, Chile

**Keywords:** angiogenic factors, periodontitis, pregnancy, spontaneous preterm birth, gingival crevicular fluid, biomarkers

## Abstract

**Background:**

Emerging evidence suggests that abnormal angiogenesis and imbalanced angiogenic factors may contribute to the development of spontaneous preterm birth (sPTB). In addition, pregnancy-related angiogenic changes and increased vascular permeability in periodontal tissues could amplify periodontal inflammation under hormonal influence.

**Objectives:**

This study aimed to evaluate the association between gingival crevicular fluid (GCF) levels of placental growth factor (PlGF) and soluble fms-like tyrosine kinase-1 (sFlt-1) and sPTB risk and to assess their correlation with periodontal disease severity during early pregnancy.

**Materials and methods:**

A prospective cohort study was conducted involving 348 pregnant women, with obstetric, clinical, and periodontal parameter assessments performed at 11–14 weeks of gestation, including probing depth (PD), clinical attachment loss (CAL), bleeding on probing (BOP), periodontal inflamed surface area (PISA), and plaque index score (PI). GCF samples were collected, and PlGF and sFlt-1 levels were measured using Magpix-Luminex® multiplex technology.

**Results:**

sPTB occurred in 3.45% (*n* = 12) of the participants. The women who had a sPTB had a significantly higher GCF PlGF/sFlt-1 ratio (*p* = 0.017) and lower sFlt-1 levels (*p* = 0.003) compared to those who had term pregnancies. A multivariate regression model combining the PlGF/sFlt-1 ratio, PI score, and first-trimester arterial blood pressure showed a predictive area under the curve of 0.78 (odds ratio 3.36, *p* = 0.008) for sPTB risk. Periodontal parameters, including PD sites >3 mm and PISA, were significantly worse in those with sPTB pregnancies (*p* = 0.032 and *p* = 0.047, respectively). Both PlGF and sFlt-1 levels were elevated in pregnant women with moderate to severe periodontitis compared to those with gingivitis or a healthy status (*p* < 0.0001), with significant positive correlations with inflammatory periodontal clinical parameters (*p* < 0.05).

**Conclusion:**

An early pregnancy imbalance of angiogenic and antiangiogenic factors in the GCF is associated with increased sPTB risk and greater periodontal inflammation. These findings suggest that angiogenic factors in the GCF may serve as promising non-invasive biomarkers for identifying women at elevated risk for sPTB.

## Introduction

Pregnancy is a unique immunological state in which the maternal immune system adapts to tolerate fetal antigens while maintaining sufficient immune defense (1). This balance comes at the cost of increased susceptibility to infections and inflammatory conditions, including periodontal disease (2–4). It is well established that the severity of periodontal disease, particularly gingival inflammation, tends to increase during pregnancy, with rates of pregnancy gingivitis ranging from 30% to 100% (5–8). Clinical signs can escalate from mild inflammation to pronounced hyperplasia, profuse bleeding, an increase in pseudo-periodontal pockets, and further progression, particularly in women with pre-existing periodontitis (5, 7, 8). Hormonal fluctuations during pregnancy—most notably elevated estrogen and progesterone—modify the subgingival microbiome, promote angiogenesis, and enhance vascular permeability, collectively amplifying local inflammatory responses in periodontal tissues (7–9).

Clinically, periodontitis is increasingly recognized not just as a local oral health issue but as a potential contributor to systemic pregnancy complications (4, 9). Recent evidence suggests a modest but consistent association between periodontitis and preterm birth (PTB). A 2024 meta-analysis of over 2.5 million participants reported that periodontitis increases the risk of PTB by approximately odds ratio (OR) 1.87 (10). Large cohort studies also support a dose–response relationship, with greater periodontal severity linked to higher PTB risk (11). Translocation of periodontal bacterial components to the maternal fetal unit, along with the systemic release of inflammatory mediators such as IL-1β, IL-6, TNF-α, and C-reactive protein (CRP), has been implicated in pre-eclampsia, gestational diabetes mellitus, fetal growth restriction, and spontaneous preterm birth (sPTB) (9–15). Gingival crevicular fluid (GCF), which bathes the periodontal sulcus, contains a dynamic mix of host immune factors, microbial byproducts, enzymes, hormones, and placental molecules, making it a promising, non-invasive biofluid for detecting systemic and local molecular changes during pregnancy (16, 17).

At the molecular level, angiogenic imbalance has been identified as a critical factor driving placental dysfunction and sPTB (18). Placental growth factor (PlGF) and its soluble antagonist, sFlt-1, are central regulators of vascular development and endothelial placental health (18, 19). While these molecules are predominantly studied in the context of placental and maternal vascular health (20), their role in peripheral tissues such as the gingiva remains largely unexplored. Angiogenesis is a fundamental component of periodontal tissue maintenance and repair, yet dysregulated angiogenic activity can exacerbate chronic inflammation, impair tissue integrity, and promote periodontal disease progression (21, 22).

The possibility that PlGF and sFlt-1 levels in the GCF reflect both local periodontal status and broader systemic vascular shifts represents an intriguing, underinvestigated avenue of research with clear clinical implications, especially considering their usefulness in the early prediction of perinatal diseases (20, 23, 24). Therefore, this study aimed to bridge the gap between periodontal and obstetric research by (1) evaluating the association between first-trimester gingival crevicular fluid PlGF/sFlt-1 ratios and the risk of sPTB, and (2) exploring the relationship between angiogenic markers in the GCF and periodontal disease severity during pregnancy. By elucidating these connections, this study seeks to support the development of novel, non-invasive oral biomarkers for the early identification of women at risk for adverse pregnancy outcomes, offering opportunities for timely intervention and promoting maternal, oral, and fetal health.

## Material and methods

### Study design and population

In total, 348 pregnant women were recruited and consecutively enrolled at the *Centro de Salud San Bernardo, Universidad de Los Andes,* in Santiago, Chile. The inclusion criteria were pregnant women between 11 and 14 weeks of gestation, aged between 18 and 38 years, who agreed to participate in the study. Each woman was examined, and demographic, clinical, physical, obstetric, and medical history were recorded. Patients were excluded if they had any associated chronic autoimmune disorder, active infections, fewer than 20 teeth, or had undergone systemic or topical antimicrobial/anti-inflammatory therapy or previous periodontal treatment for the previous 3 months. Pregnant women were followed from the entry date until baby delivery and sPTB was the outcome variable. Women who were diagnosed with spontaneous preterm birth (spontaneous delivery before 37 weeks) were assigned to the sPTB group. Otherwise, pregnant women were assigned to the term delivery group. After periodontal evaluation and collection of GCF samples, all pregnant women received case-specific periodontal diagnosis, oral hygiene instructions, and professional supragingival scaling. The study protocol was clearly explained to all the participants, who signed an informed consent approved by the Ethics Committee of the Universidad de los Andes (#CEC2021021, April 23, 2021). Registration was made under the Declaration of Helsinki, as amended in 2013.

The main outcome variable was the development of sPTB and its association with the concentrations of angiogenic/antiangiogenic factors in the gingival crevicular fluid samples of pregnant women at 11–14 weeks of gestation. The secondary outcomes included the relationship between the periodontal diagnosis and periodontal clinical parameters and the concentrations of angiogenic/antiangiogenic factors in the gingival crevicular fluid. The studied variables were age, weight, height, body mass index (BMI), periodontal clinical parameters [probing depth (PD), clinical attachment loss (CAL), bleeding on probing (BOP), and periodontal pockets >3 mm], periodontal inflamed surface area (PISA), plaque index score, number of teeth, smoking history, current smoker, systolic blood pressure, diastolic blood pressure, median arterial blood pressure, and sFlt-1 and PlGF concentrations in gingival crevicular fluid.

### Definitions

Spontaneous preterm birth was defined based on the WHO definition as babies born alive by spontaneous causes before 37 weeks of pregnancy are completed. Periodontitis was defined as stated by the 2017 World Workshop as interdental CAL detectable in ≥2 non-adjacent teeth, or buccal or oral CAL ≥ 3 mm with pocketing >3 mm detectable in ≥2 teeth (25). Gingivitis was defined as subjects who did not exhibit PD greater than or equal to 3 mm without CAL or patients with CAL (reduced periodontium, non-periodontitis patients), and positive BOP at ≥10% of the probing sites. Gingival health was defined as <10% bleeding at the probing sites with rank ≤ 3 mm (26).

### Sample size estimation

This is a secondary analysis of an ongoing cohort involving 348 pregnant women at 11–14 gestation weeks. The estimated sample size for a previous cohort study (not yet published), whose main outcome was preeclampsia development and its association with angiogenic/antiangiogenic markers in gingival crevicular samples, considered a preeclampsia prevalence of 6.4%, a significance level of 5%, and a power of 80% for a two-sided test, and a loss of 10%. A total sample size of 358 pregnant women was estimated for this study.

### Periodontal clinical examination

A complete periodontal evaluation was performed by a single calibrated operator at 11–14 weeks' gestation using a North Carolina (Hu-friedy®) periodontal probe. The following measures were recorded at six sites per tooth: PD, CAL, BOP, visible dental plaque accumulation along the gingival margin (recorded as presence or absence), and PISA (27).

### GCF samples and elution protocol

GCF samples were obtained by placing paper strips (Periopaper®, ProFlow, Amityville, New York, USA) into the gingival sulcus or pocket for 30 s by a trained periodontist. In each patient, pooled GCF samples from each mouth quadrant were obtained, with one paper strip per quadrant and four per patient. The selection of sampled sites was made based on the periodontal diagnosis. If the pregnant woman had gingivitis, the teeth affected by gingivitis were selected. The same criteria were used to select the healthy and periodontitis sites. The collected GCF was subsequently eluted from the paper strips. Then, 160 μL of elution buffer was added, composed of Tris-HCl 0.5 M pH 7.5 (Tris-HCl ULTROL Grade, Merck, Darmstadt, Germany), NaCl 2 M (Sodium chloride, ReagentPlus ≥99%, Sigma-Aldrich, MO, USA), CaCl_2_ 250 mM (Calcium chloride, anhydrous, powder ≥9, Sigma-Aldrich, MO, USA), and Triton X-100 (25%) (Triton X-100, Sigma-Aldrich, MO, USA), in the presence of an EDTA-free protease inhibitor (cOmplete™, Mini, EDTA-free Protease I, Roche, Basel, Switzerland). The paper strips were vortexed for 30 s at maximum speed. Samples were then incubated for 30 min on ice, centrifuged for 5 min at 12,000 × *g* at 4°C, and the supernatant was eluted and kept on ice. The samples were transferred into 1.5 mL microcentrifuge tubes and the process was repeated. The new eluate was rescued and mixed with the eluate that was kept on ice. The final 320 μL of the eluted samples were stored at −80°C for further analysis.

### Luminex assay

Processed samples were analyzed using a Multiplex Bead Immunoassay (Human Magnetic Luminex Assay®, R&D Systems, Minneapolis, MN, USA) according to the manufacturer's instructions to quantify both the angiogenic markers, sFlt-1 and PlGF. Protein concentrations were measured using a digital platform (Magpix, Millipore, St. Charles, MO, USA) and analyzed using MILLIPLEX AnalystR software® (v5.1, Viagene Tech, Carlisle, MA, USA). All reagents and samples (previously eluted) were at room temperature before analysis. All samples were analyzed in duplicate.

### Statistical analysis

The normality of the quantitative data was assessed using the Shapiro–Wilk test. Data with a normal distribution were summarized using the mean and standard deviation (SD), while non-normally distributed data were described using median and interquartile range (IQR). Groups were defined according to the outcome (sPTR or term delivery). Differences between groups were assessed using the Mann–Whitney *U*-test or the Kruskal–Wallis test. Correlation coefficients were estimated using Spearman's test. Using two multiple logistic regression models, the crude OR with a 95% confidence interval (CI) was determined to assess the likelihood of developing sPTB. The selection of the variables considered the clinical approach, the bivariate analysis, and the performance of the regression models. Therefore, plaque index score and median arterial blood pressure were included in the model because the predictive performance of both biomarkers (PIGF/sFlt-1 ratio) increased significantly when they were added to the regression models. The first model determined the relationship between sFlt-1 concentration in GCF, median arterial blood pressure, and plaque index score and the main outcome. The second model assessed the likelihood of developing sPTB in relation to first-trimester PlGF/sFlt-1 concentrations in GCF, median arterial blood pressure, and plaque index score. Hosmer and Lemeshow’s goodness-of-fit test was performed to evaluate the fit of both models. The discrimination performance of this model was evaluated through the area under the curve (AUC) of the receiver operating characteristic (ROC) curve. The optimal cutoff points were estimated to assess the highest sensitivity and specificity. Statistical significance was defined as *p* ≤ 0.05. Data were analyzed using STATA v18 software (StataCorp, College Station, TX, USA), and the graphs were created using GraphPad Prism version 10.1.1 for macOS (GraphPad Software, Boston, MA, USA, http://www.graphpad.com) and STATA.

## Results

### Clinical data and maternal characteristics

Demographic and clinical data are presented in Table 1. A total of 348 pregnant women were enrolled in this cohort study. Of those, 12 women (3.45%) had sPTB. At the first trimester screening, systolic arterial pressure and median arterial pressure were significantly different in those who had sPTB and term delivery pregnancies (*p* = 0.038 and *p* = 0.044, respectively). Regarding the participants’ periodontal diagnoses, 8.3% were clinically periodontally healthy, 24.1% had gingivitis, 52.2% had stage I/II periodontitis, and 15.2% had stage III/IV periodontitis. Probing depth, percentage of periodontal pockets >3 mm, and PISA were higher in those who had spontaneous preterm birth pregnancies compared to those who had healthy term deliveries (*p* = 0.050, *p* = 0.032, and *p* = 0.047, respectively) (Table 2).

**Table 1:** Demographic and clinical characteristics of the pregnant women (at 11–14 weeks of gestation) according to the spontaneous preterm birth (sPTB) and healthy term delivery outcomes.

Variable		sPTB (*n* = 12)	Healthy term delivery (*n* = 336)	*p*-Value^a^
Age (years)	Mean (SD)	27.42 (5.05)	25.71 (5.36)	0.207
	Median (IQR)	27.50 (8.00)	25.00 (9.00)	
	Min/max	19/36	18/ 38	
Weight (kg)	Mean (SD)	73.00 (22.52)	69.71 (14.28)	0.979
	Median (IQR)	64.50 (32.00)	68.00 (19.00)	
	Min/max	51/128	40/118	
Height (cm)	Mean (SD)	158.58 (5.76)	157.48 (10.23)	0.715
	Median (IQR)	158.50 (6.00)	158 (7.00)	
	Min/max	148/168	1.58/175	
BMI	Mean (SD)	28.82 (7.57)	27.81 (5.19)	0.853
	Median (IQR)	27.63 (9.50)	27.00 (7.24)	
	Min/max	20/46	16.87/49	
Systolic arterial blood pressure (mmHg )	Mean (SD)	112.25 (11.20)	104.54 (11.48)	**0**.**038**
	Median (IQR)	113.50 (20.00)	102.00 (10.00)	
	Min/max	90/125	80/142	
Diastolic arterial blood pressure (mmHg )	Mean (SD)	66.83 (7.16)	63.58 (8.16)	0.120
	Median (IQR)	69.00 (11.00)	60.00 (10.00)	
	Min/max	56/80	40/92	
Median arterial blood pressure (mmHg )	Mean (SD)	88.92 (7.94)	84.05 (8.62)	**0**.**044**
	Median (IQR)	91.00/11.00	84.00 (10.00)	
	Min/max	75/100	60/113	
Smoking history		*n* (cell %)	*n* (cell %)	*p*-Value^b^
	Yes	7 (2.01)	164 (47.13)	0.569
	No	5 (1.44)	172 (49.43)	
Current smoker	Yes	2 (0.57)	66 (18.97)	0.100
	No	10 (2.87)	270 (77.59)	
Periodontal diagnosis	Healthy	3 (0.86)	26 (7.47)	0.072
	Gingivitis	4 (1.15)	80 (22.99)	
	Stage I/II periodontitis	5 (1.44)	177 (50.86)	
	Stage III/IV periodontitis	0 (0.00)	53 (15.23)	

Bold values mean *p* < 0.05.

sPTB, spontaneous preterm birth; BMI, body mass index.

^a^ Mann–Whitney test.

^b^ Chi-squared test. Significance level *p* ≤ 0.05.

**Table 2:** Periodontal clinical variables of the pregnant women (at 11–14 weeks of gestation) according to the spontaneous preterm birth (sPTB) and healthy term delivery outcomes.

Variable		sPTB (*n* = 12)	Healthy term delivery (*n* = 336)	*p*-Value^a^
Number of teeth	Mean (SD)	26.58 (2.32)	25.67 (4.21)	0.296
	Median (IQR)	27.00 (2.00)	26.50 (1.50)	
	Min/max	16/32	14/31	
Periodontal pockets >3 mm (%)	Mean (SD)	25.31 (21.49)	13.50 (15.24)	**0**.**032**
	Median (IQR)	22.00 (28.00)	8 (17.50)	
	Min/max	0/104	0/49	
Pocket depth (mm)	Mean (SD)	2.63 (0.48)	2.35 (0.43)	**0**.**050**
	Median (IQR)	2.60 (0.60)	2.35 (0.60)	
	Min/max	1.4/4.4	1.5/3.1	
Clinical attachment loss (mm)	Mean (SD)	2.17 (2.22)	1.87 (0.50)	0.372
	Median (IQR)	1.90 (0.70)	1.90 (0.70)	
	Min/max	0.9/41	1.2/2.9	
Bleeding on probing (%)	Mean (SD)	59.79 (26.04)	57.92 (21.28)	0.913
	Median (IQR)	60.00 (44.00)	50.00 (32.50)	
	Min/max	60/100	28/100	
Plaque index score (%)	Mean (SD)	62.35 (29.48)	68.08 (27.22)	0.498
	Median (IQR)	67.00 (53.00)	73.00 (50.00)	
	Min/max	6/100	30/100	
PISA (mm^2^)	Mean (SD)	900.14 (508.18)	598.68 (302.12)	**0**.**047**
	Median (IQR)	839.80 (767.40)	629.35 (515.25)	
	Min/max	**111.8/2,604.3**	**161.7/1,048.1**	

Bold values mean *p* < 0.05.

sPTB, spontaneous preterm birth; PD, probing depth; CAL, clinical attachment loss; BOP, bleeding on probing; PI, plaque index score; PISA, periodontal inflamed surface area.

^a^ Mann–Whitney test, significance level *p* ≤ 0.05.

### First-trimester assessment of GCF angiogenic factors, spontaneous preterm birth, and maternal periodontitis

**Figure 1:**
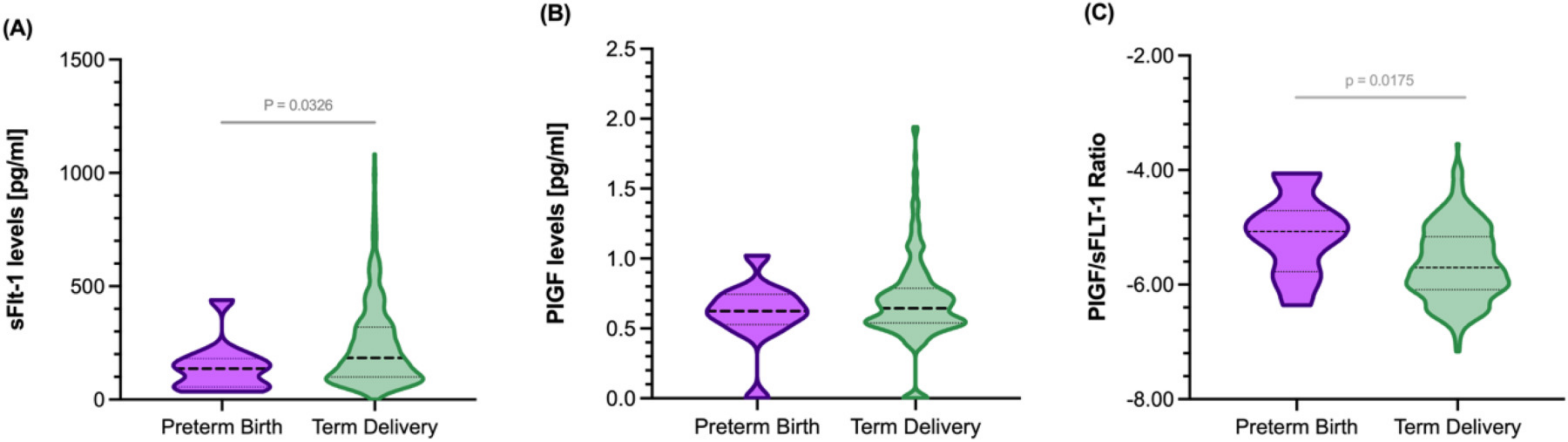
Gingival crevicular fluid concentrations of sFLt-1 and PlGF and their respective ratio in pregnant women at 11–14 weeks gestation in the studied population according to preterm birth development (PTB). **(A)** sFlt-1 concentration, **(B)** PlGF concentration, and **(C)** PlGF/sFlt-1 ratio.

At 11–14 weeks, the analysis of the PlGF concentration within GCF revealed a median value of 0.62 pg/mL (IQR 0.19) among women who subsequently experienced spontaneous preterm birth, compared to a median value of 0.65 pg/mL (IQR 0.24) in pregnant women who had healthy term deliveries (*p* *=* 0.577). Conversely, the levels of GCF-sFlt-1 were decreased in the women who had a sPTB compared to those who had healthy term delivery pregnancies [median value of 136.78 pg/mL (IQR 114.05) vs.183.44 pg/mL (IQR 220.30), respectively, *p* = 0.032]. Furthermore, the PlGF/sFlt-1 ratio was significantly increased at 11–14 weeks in the pregnant women who later had a sPTB (*p* = 0.017) (Figure 1).

**Figure 2:**
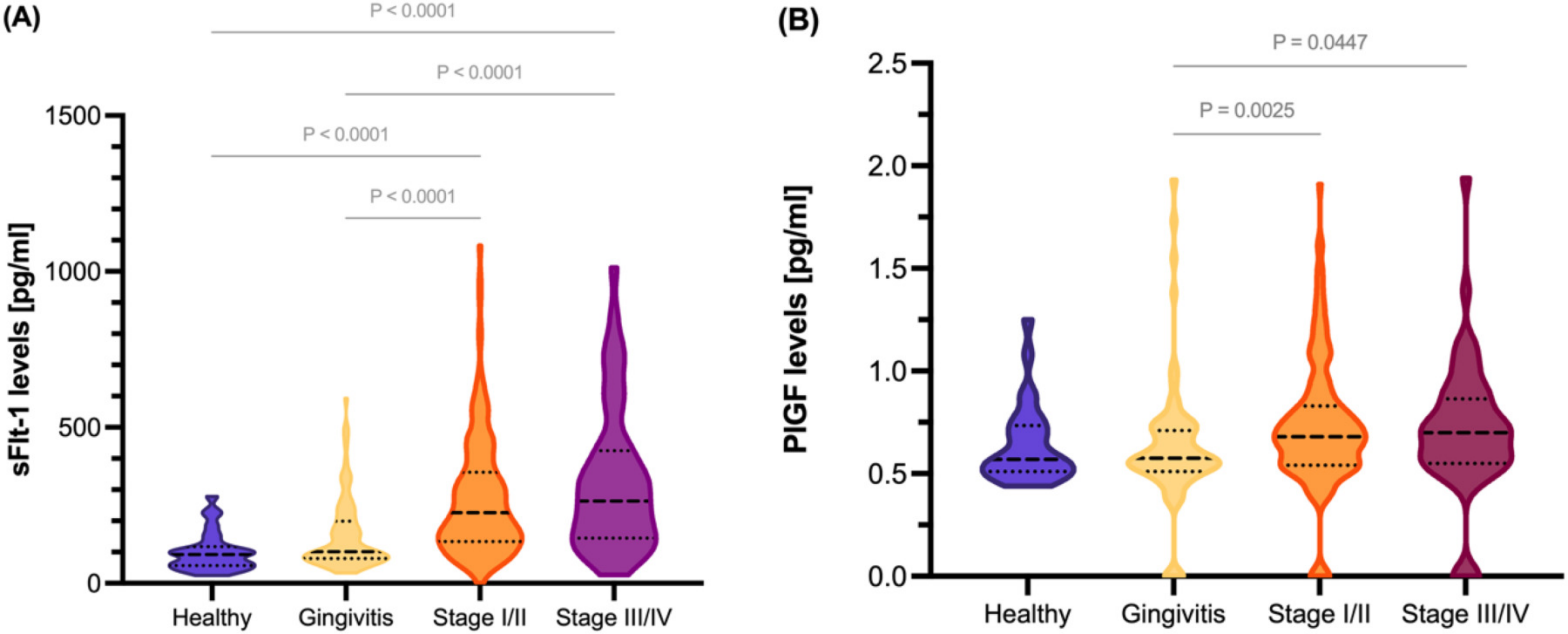
Gingival crevicular fluid concentrations of sFlt-1 and PlGF in pregnant women at 11–14 weeks of pregnancy in the studied population and their respective ratios by periodontal diagnosis status. **(A)** sFlt-1 concentration and **(B)** PlGF concentration.

**Figure 3:**
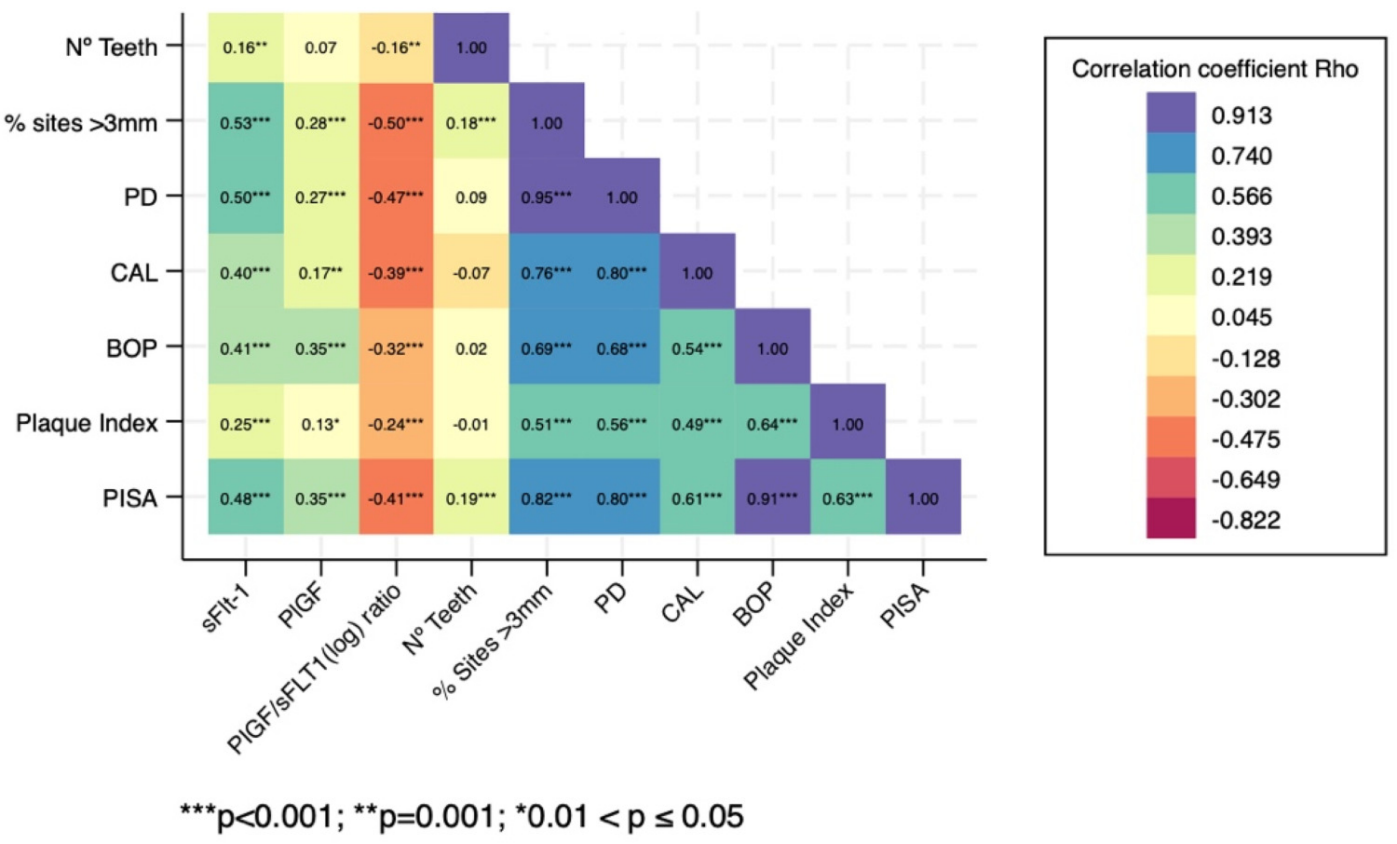
Correlation heatplot between gingival crevicular fluid concentrations of sFlt-1 and plGF and their respective ratios at 11–14 weeks of pregnancy and the periodontal clinical inflammatory parameters of the pregnant women in the studied population. PD, probing depth; CAL, clinical attachment loss; BOP, bleeding on probing; PISA, periodontal inflamed surface area.

When analyzing the concentrations of both angiogenic factors in GCF according to the periodontal diagnosis, sFlt-1 concentrations were statistically significantly increased in those with periodontitis compared to those with gingivitis and healthy pregnancies (*p* *<* 0.0001) (Figure 2). The median concentration of sFlt-1 was nearly three times higher in patients with stage III/IV periodontitis [263.53 pg/mL, (IQR 275.96)] compared to those who were periodontally clinically healthy [91.90 pg/mL, (IQR 48.51)]. PlGF levels were significantly increased in those with periodontitis stages I/II (*p* *=* 0.002) and periodontitis stages III/IV (*p* *=* 0.044) compared to those with a gingivitis diagnosis (Figure 2). Spearman’s correlation between the clinical inflammatory periodontal parameters and the concentrations of both angiogenic markers was also determined (Figure 3). PlGF levels were positively associated with all periodontal parameters (*p* ≤ *0.05*). The highest correlation was found for PISA and BOP. A moderate association between sFlt-1 and the percentage of sites > 3 mm, PD, and PISA was observed. Detailed correlation results are shown in Figure 3.

### Diagnostic predictive performance of GCF-derived angiogenic factors

**Figure 4:**
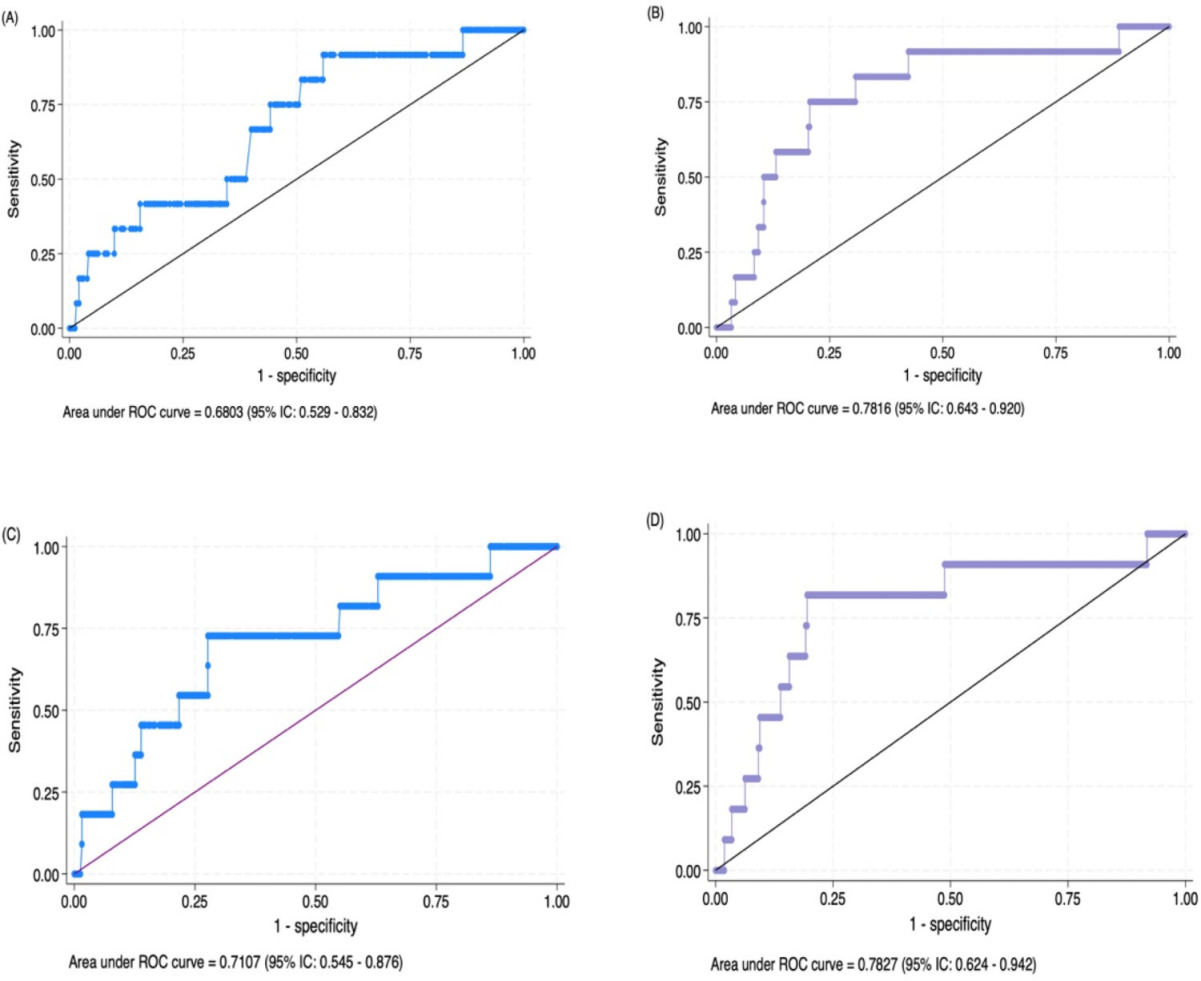
**(A)** Area under the receiver operating characteristic curve (AUC-ROC) of the simple logistic regression analysis including the sFlt-1 concentration in the GCF. **(B)** AUC-ROC curve of the model including the sFlt-1 concentration in the GCF, plaque index score, and first-trimester arterial blood pressure. **(C)** AUC-ROC curve of the model including the PlGF/sFlt-1 ratio. **(D)** AUC-ROC curve of the model including the PlGF/sFlt-1 ratio, plaque index score, and first-trimester arterial blood pressure for the prediction of sPTB risk.

The performance of single and multiparametric models (Table 3) was also explored. Two single logistic regression models (Models A and C) were conducted, one with sFlt-1 and the other with the PlGF/sFlt-1 ratio as variables to predict sPTB. The other two models (B and D) were adjusted by median arterial blood pressure and the plaque index score due to the results of the bivariate analysis, the clinical relevance, and the increased ability to predict the likelihood of sPTB when both biomarkers were included in the models. The multiple logistic regression model that included sFlt-1, plaque index score, and median arterial blood pressure (Table 3, Model B) showed an association between sFlt-1 and the likelihood of sPTB [OR: 0.99 (CI 0.01–0.00), *p* = 0.049]. The area under the ROC curve observed for this model was 0.78 (95% CI: 0.643–0.920), resulting in a predictive model for spontaneous preterm birth risk with a specificity of 79.4% and a sensitivity of 75%. The model correctly classified 79.25% of the women who had a sPTB and had a Hosmer and Lemeshow's goodness-of-fit test value of *p* = 0.624 (Figure 4B). In addition, the model that considered PlGF/sFlt-1 ratio, plaque index score and median arterial blood pressure (Table 3, Model D) showed an association between PlGF/sFlt-1ratio and sPTB [OR: 3.36 (CI 1.37–8.26), *p* = 0.008] and the area under the ROC curve observed was 0.78 (95% CI: 0.624–0.942). This model correctly classified 70.23% of the women who had a sPTB with a specificity of 70.05% and sensitivity of 75% (Figure 4D).

**Table 3:** Logistic regression models for the likelihood of sPTB at 11–14 weeks of pregnancy.

Regression model	Odds ratio (OR)	95% confidence interval	*p*-Value
Model A			
sFlt-1 concentration in GCF	0.994	0.989–1.001	0.073
Model B			
sFlt-1 concentration in GCF	**0**.**994**	0.989–0.999	**0**.**049**
Plaque index score	1.01	0.0992–1.034	0.242
Median arterial blood pressure	1.06	0.998–1.137	0.058
Model C			
-PlGF/sFlt-1 ratio in GCF	**3**.**22**	1.30–7.96	**0**.**011**
Model D			
PlGF/sFlt-1 ratio in GCF	**3**.**36**	1.36–8.26	**0**.**008**
Plaque index score	1.01	0.99–1.03	0.422
Median arterial pressure	1.06	0.99–1.14	0.102

Model A: Simple logistic regression analysis including sFlt-1 concentration in GCF. Model B: Multiple logistic regression model including sFlt-1 concentration in GCF, plaque index score, and first-trimester arterial blood pressure. Model C: Simple logistic regression model including the PIGF/sFlt-1 ratio. Model D: Multiple logistic regression model including the PIGF/sFlt-1 ratio, plaque index score, and first-trimester arterial blood pressure.

Bold values mean *p* < 0.05.

## Discussion

To our knowledge, this is the first study to analyze an imbalance of angiogenic factors in gingival crevicular fluid as early predictors of spontaneous preterm birth risk and their association with periodontal diagnosis during early pregnancy. The main conclusions that may be drawn from this study are as follows: (1) there was an imbalance in gingival crevicular fluid angiogenic factors, particularly sFlt-1, in pregnant women with periodontitis compared to those with gingivitis and those who were periodontally healthy; (2) sFlt-1 was reduced in the pregnant women who developed spontaneous preterm birth since early pregnancy; (3) sFlt-1 and the PlGF/sFlt-1 ratio, when combined with the plaque index score and median arterial blood pressure in multiparametric regression models, have potential utility as oral biomarkers for predicting the risk of preterm birth, with a reasonably AUC-ROC curve (0.78), indicating a potential association between periodontal health status and alterations in angiogenic factor levels during pregnancy.

Pregnancy promotes the expression of a proinflammatory and immunosuppressing phenotype (1–3). It is believed that this immune-inflammatory shift, accompanied by high levels of pregnancy-related hormones and combined with an impaired neutrophil function and a proangiogenic environment, may enhance gingival inflammation and the severity of periodontal disease (22, 28, 29). Furthermore, pregnancy hormones could favor a proangiogenic state by augmenting the amount of angiogenic factors and protecting macrophages from apoptosis, thus prolonging the angiogenic effect in periodontal tissues (29). We propose that sFlt-1 and PlGF are potentially involved in the gingival inflammation observed during pregnancy. In this sense, in the present study, we observed an increased expression of sFlt-1 and PlGF in pregnant women with periodontitis compared to those with gingivitis or healthy pregnant women. Furthermore, a correlation between an increase in both angiogenic factors and periodontal inflammatory clinical parameters, such as probing depth, clinical attachment loss, and bleeding on probing, was also observed.

PlGF is a member of the VEGF family, and it binds to Flt-1, VEGF-R1, or its soluble form (30). PlGF was originally identified in the placenta, contributing to trophoblastic growth and differentiation, but is now known to be expressed by multiple organs, such as the heart, lungs, thyroid, and muscles (31, 32). The PlGF functions described to date include acting as a powerful chemoattractant for monocytes and macrophages and contributing to the growth of vessels and the migration and survival of endothelial and bone marrow progenitor cells (33, 34). The plasma expression of PlGF is low in most healthy tissues but can be significantly increased in inflammatory conditions (33–35). It has also been linked to increased toll-like receptor signaling activity, producing a prolonged proinflammatory state, with the release of various proinflammatory cytokines and acute phase reactants (35). Flt-1 acts as a receptor for PlGF (36), and it has two forms: the transmembrane and its soluble form, which lacks cytoplasmic and transmembrane domains (37). Its main sources are endothelial cells, monocytes, and the placenta, and it prevents angiogenesis (38). Different studies suggest that altered levels of PlGF and sFlt-1 may contribute to impaired placental function, oxidative stress, and endothelial dysfunction, potentially leading to the onset of preterm labor (20, 23). An imbalance between the two angiogenic factors has been associated with various pregnancy complications, including sPTB (39, 40). Previously, we demonstrated that increased PlGF concentration in GCF was associated with the subsequent risk of gestational diabetes mellitus (GDM), where PlGF levels in GCF combined with first-trimester fasting glycemia were a predictor of GDM risk (AUC-ROC: 89%) (41). Moreover, sFlt-1 has also been associated with an increased risk of pre-eclampsia, with increased concentrations of sFlt-1 in the saliva and GCF samples of pre-eclamptic women compared to those who had a term delivery and healthy pregnancies (42).

We speculate that an imbalance in the PlGF/sFlt-1 ratio leads to increased vascular permeability and periodontal inflammation since early pregnancy. As an example, the binding of PlGF to Flt-1 in patients with rheumatoid arthritis stimulated the production of proinflammatory cytokines, including TNF-α and IL-6 in monocytes, enhancing inflammation (43). There is limited evidence regarding the direct role of the two angiogenic factors in pregnant women with periodontal disease. However, it has been described that the Flt-1 gene is highly upregulated in periodontitis-associated fibroblasts (21). In addition, Horton et al. investigated the sFlt-1 levels in maternal plasma in pregnant women before 26 weeks' gestation, but found no differences regarding periodontitis severity (44). Nevertheless, the specific mechanisms by which sFlt-1 and PlGF impact the severity of periodontal disease physiopathology are still being investigated, and the precise implications of the two angiogenic factors in the context of maternal periodontitis during pregnancy and the risk of adverse pregnancy outcomes remain to be fully elucidated.

The relationship between the severity of maternal periodontitis and PTB has been evaluated in previous studies, with the relative risk of PTB ranging from 1.6 to 3.4 (45–53). In this sense, a study conducted in 1,020 pregnant women before the 26th week of gestation found an RR of 1.6 (95% CI: 1.1–3.3) for moderate to severe periodontitis and PTB (45). Another study in over 1,000 pregnant women showed that those with periodontitis and preeclampsia had a higher risk of PTB with a hazard ratio of 4.11 and 11.00, respectively, when they were affected by moderate to severe periodontitis (46). In contrast, other prospective studies have not shown associations between periodontitis and PTB (47, 48). In this study, we did not find a direct association between maternal periodontitis and sPTB. However, we observed an increase in the severity of periodontal clinical parameters, including probing depth, percentage of periodontal pockets, and PISA, with more inflammation since the beginning of pregnancy in the women who subsequently had a sPTB compared to those who had a healthy term delivery.

Regarding their usefulness as early biomarkers for the prediction of PTB, the evidence indicates that plasmatic PlGF and sFlt-1 can be used separately, or the PlGF/sFlt-1 and sFlt-1/PlGF ratios can be used (18–20). Both ratios reflect an imbalance of angiogenic factors and vascular pathology in the placental tissues (39, 40). In the present study, we observed a similar trend, with an increased PlGF/sFlt-1 ratio found in the first-trimester GCF samples from pregnant women who had a sPTB. We propose that assessing the angiogenic vascular status in GCF through liquid biopsies in the first trimester of pregnancy represents a novel and non-invasive approach, furthering our understanding of the association between periodontitis and PTB and of early prediction of sPTB risk. Furthermore, the understanding of the intricate balance between angiogenic factors in periodontal tissues will be crucial for elucidating the relationship between periodontal disease severity and adverse pregnancy outcomes.

A critical limitation of our study is the small number of sPTB cases observed (*n* = 12, 3.4% of the cohort), which may affect the statistical power, precision of the estimates, and generalizability of the results. Although our multiparametric regression models yielded promising AUC values, the small event number raises the risk of model overfitting and wide confidence intervals. Future studies with larger and more diverse populations are essential to validate the diagnostic performance of GCF angiogenic markers in predicting sPTB. Ideally, *a priori* or *post hoc* power calculations should be incorporated in subsequent studies to formally assess whether the sample size provides sufficient power to detect clinically meaningful associations. Importantly, while we report significant associations between GCF angiogenic markers, maternal periodontal status, and sPTB risk, our findings should not be interpreted as evidence of causality. The observed relationships may be influenced by unmeasured confounding factors, and the cross-sectional nature of the early pregnancy sampling limits the ability to infer directionality. Future mechanistic and longitudinal studies are warranted to unravel the causal pathways linking local periodontal angiogenic changes to systemic pregnancy outcomes. Specifically, investigating the biological mechanisms, such as the potential translocation of inflammatory mediators or bacterial products from periodontal tissues to the systemic circulation, or the systemic vascular adaptations that may concurrently affect placental and periodontal microenvironments, would enrich our understanding of how oral health influences pregnancy outcomes.

## Conclusions

Angiogenic factors could play a significant role in identifying women at risk of spontaneous preterm birth and may exacerbate periodontal inflammation during pregnancy. These findings should be further validated in subsequent prospective cohort studies that may yield valuable insights into oral biomarkers for the prediction of preterm birth and the pathophysiology of pregnancy periodontal disease and potentially pave the way for novel diagnostic and therapeutic strategies aimed at modulating angiogenic responses and inflammation within the periodontal microenvironment.

## Data Availability

The original contributions presented in the study are included in the article/Supplementary Material. Further inquiries can be directed to the corresponding author.

## References

[1] Mor G Cardenas I. The immune system in pregnancy: a unique complexity. Am J Reprod Immunol. (2010 Jun) 63(6):425–33. 10.1111/j.1600-0897.2010.00836.x 20367629 PMC3025805

[2] Mor G Koga K. Macrophages and pregnancy. Reprod Sci. (2008) 15:435–6. 10.1177/1933719108317253 18579852

[3] Dekel N Gnainsky Y Granot I Mor G. Inflammation and implantation. Am J Reprod Immunol. (2010) 63:17–21. 10.1111/j.1600-0897.2009.00792.x 20059465 PMC3025807

[4] Armitage GC. Bi-directional relationship between pregnancy and periodontal disease. Periodontol 2000. (2013) 61(1):160–76. 10.1111/j.1600-0757.2011.00396.x 23240948

[5] Figuero E Carrillo-de-Albornoz A Martín C Tobías A Herrera D. Effect of pregnancy on gingival inflammation in systemically healthy women: a systematic review. J Clin Periodontol. (2013) 40(5):457–73. 10.1111/jcpe.12053 23557432

[6] Morelli EL Broadbent JM Leichter JW Thomson WM. Pregnancy, parity and periodontal disease. Aust Dent J. (2018) 63(3):270–8. 10.1111/adj.12623 29770451

[7] González-Jaranay M Téllez L Roa-López A Gómez-Moreno G Moreu G. Periodontal status during pregnancy and postpartum. PLoS One. (2017) 12(5):e0178234. 10.1371/journal.pone.0178234 28538740 PMC5438174

[8] Xie Y Xiong X Elkind-Hirsch KE Pridjian G Maney P Delarosa RL Change of periodontal disease status during and after pregnancy. J Periodontol. (2013) 84(6):725–31. 10.1902/jop.2012.120235 22873653

[9] Madianos PN Bobetsis YA Offenbacher S. Adverse pregnancy outcomes (APOs) and periodontal disease: pathogenic mechanisms. J Periodontol. (2013) 84(4 Suppl):S170–80. 10.1902/jop.2013.1340015 23631577

[10] Castaño-Suárez L Paternina-Mejía GY Vásquez-Olmos LD Rodríguez-Medina C Botero JE. Linking periodontitis to adverse pregnancy outcomes: a comprehensive review and meta-analysis. Curr Oral Health Rep. (2024) 11:125–37. 10.1007/s40496-024-00371-6

[11] Ren H Du M. Role of maternal periodontitis in preterm birth. Front Immunol. (2017) 8:139. 10.3389/fimmu.2017.00139 28243243 PMC5303728

[12] Figuero E Han YW Furuichi Y. Periodontal diseases and adverse pregnancy outcomes: mechanisms. Periodontol 2000. (2020) 83(1):175–88. 10.1111/prd.12295 32385886

[13] Bobetsis YA Graziani F Gürsoy M Madianos PN. Periodontal disease and adverse pregnancy outcomes. Periodontol 2000. (2020) 83(1):154–74. 10.1111/prd.12294 32385871

[14] Machado V Ferreira M Lopes L Mendes JJ Botelho J. Adverse pregnancy outcomes and maternal periodontal disease: an overview on meta-analytic and methodological quality. J Clin Med. (2023) 12(11):3635. 10.3390/jcm12113635 37297830 PMC10253546

[15] Bobetsis YA Ide M Gürsoy M Madianos PN. Periodontal diseases and adverse pregnancy outcomes. Present and future. Periodontol 2000. (2023). 10.1111/prd.12486 37149740

[16] Barros SP Williams R Offenbacher S Morelli T. Gingival crevicular fluid as a source of biomarkers for periodontitis. Periodontol 2000. (2016) 70(1):53–64. 10.1111/prd.12107 26662482 PMC4911175

[17] Buduneli N Bıyıkoğlu B Kinane DF. Utility of gingival crevicular fluid components for periodontal diagnosis. Periodontol 2000. (2024) 95(1):156–75. 10.1111/prd.12595 39004819

[18] Levine RJ Maynard SE Qian C Lim KH England LJ Yu KF Circulating angiogenic factors and the risk of preeclampsia. N Engl J Med. (2004) 350:672–83. 10.1056/NEJMoa031884 14764923

[19] Kusanovic JP Romero R Chaiworapongsa T Erez O Mittal P Vaisbuch E A prospective cohort study of the value of maternal plasma concentrations of angiogenic and anti-angiogenic factors in early pregnancy and mid trimester in the identification of patients destined to develop preeclampsia. J Matern Fetal Neonatal Med. (2009) 22:1021–38. 10.3109/14767050902994754 19900040 PMC3427777

[20] Romero R Nien JK Espinoza J Todem D Fu W Chung H A longitudinal study of angiogenic (placental growth factor) and anti-angiogenic (soluble endoglin and soluble vascular endothelial growth factor receptor-1) factors in normal pregnancy and patients destined to develop preeclampsia and deliver a small for gestational age neonate. J Matern Fetal Neonatal Med. (2008) 21:9–23. 10.1080/14767050701830480 18175241 PMC2587364

[21] Ohshima M Yamaguchi Y Ambe K Horie M Saito A Nagase T Fibroblast VEGF-receptor 1 expression as molecular target in periodontitis. J Clin Periodontol. (2016) 43(2):128–37. 10.1111/jcpe.12495 26932322

[22] Yuan K Lin MT. The roles of vascular endothelial growth factor and angiopoietin-2 in the regression of pregnancy pyogenic granuloma. Oral Dis. (2004) 10(3):179–85. 10.1046/j.1601-0825.2003.00997.x 15089929

[23] Chen W Wei Q Liang Q Song S Li J. Diagnostic capacity of sFlt-1/PlGF ratio in fetal growth restriction: a systematic review and meta-analysis. Placenta. (2022) 127:37–42. 10.1016/j.placenta.2022.07.020 35952596

[24] Romero R Jung E Chaiworapongsa T Erez O Gudicha DW Kim YM Toward a new taxonomy of obstetrical disease: improved performance of maternal blood biomarkers for the great obstetrical syndromes when classified according to placental pathology. Am J Obstet Gynecol. (2022) 227(4):615.e1–25. 10.1016/j.ajog.2022.04.015 36180175 PMC9525890

[25] Caton JG Armitage G Berglundh T Chapple ILC Jepsen S Kornman KS A new classification scheme for periodontal and peri-implant diseases and conditions—introduction and key changes from the 1999 classification. J Periodontol. (2018) 89(Suppl 1):S1–8. 10.1002/JPER.18-0157 29926946

[26] Chapple ILC Mealey BL Van Dyke TE Bartold PM Dommisch H Eickholz P Periodontal health and gingival diseases and conditions on an intact and a reduced periodontium: consensus report of workgroup 1 of the 2017 world workshop on the classification of periodontal and peri-implant diseases and conditions. J Periodontol. (2018) 89(Suppl 1):S74–84. 10.1002/JPER.17-0719 29926944

[27] Nesse W Abbas F van der Ploeg I Spijkervet FK Dijkstra PU Vissink A. Periodontal inflamed surface area: quantifying inflammatory burden. J Clin Periodontol. (2008) 35(8):668–73. 10.1111/j.1600-051X.2008.01249.x 18564145

[28] Gürsoy M Könönen E Gürsoy UK Tervahartiala T Pajukanta R Sorsa T. Periodontal status and neutrophilic enzyme levels in gingival crevicular fluid during pregnancy and postpartum. J Periodontol. (2010) 81(12):1790–6. 10.1902/jop.2010.100147 20831370

[29] Yuan K Wing L-YC Lin MT. Pathogenetic roles of angiogenic factors in pyogenic granulomas in pregnancy are modulated by female sex hormones. J Periodontol. (2002) 73(7):701–8. 10.1902/jop.2002.73.7.701 12146528

[30] Dewerchin M Carmeliet P. PlGF: a multitasking cytokine with disease-restricted activity. Cold Spring Harb Perspect Med. (2012) 2(8):a011056. 10.1101/cshperspect.a011056 22908198 PMC3405829

[31] Yano K Okada Y Beldi G Shih S-C Bodyak N Okada H Elevated levels of placental growth factor represent an adaptive host response in sepsis. J Exp Med. (2008) 205(11):2623–31. 10.1084/jem.20080398 18852292 PMC2571936

[32] Kang M Jeong J Lee J Park S Sung Y Choi M Placental growth factor (PlGF) is linked to inflammation and metabolic disorders in mice with diet-induced obesity. Endocr J. (2018) 65(4):437–47. 10.1507/endocrj.EJ17-0363 29434073

[33] Carnevale D Lembo G. Placental growth factor and cardiac inflammation. Trends Cardiovasc Med. (2012) 22(8):209–12. 10.1016/j.tcm.2012.07.022 22925712

[34] Li X Jin Q Yao Q Zhou Y Zou Y Li Z Placental growth factor contributes to liver inflammation, angiogenesis, fibrosis in mice by promoting hepatic macrophage recruitment and activation. Front Immunol. (2017) 8:801. 10.3389/fimmu.2017.00801 28744285 PMC5504098

[35] Newell LF Holtan SG Yates JE Pereira L Tyner JW Burd I PlGF enhances TLR-dependent inflammatory responses in human mononuclear phagocytes. Am J Reprod Immunol. (2017) 78(4):e12709. 10.1111/aji.12709 PMC5915625 28635072

[36] Luttun A Tjwa M Carmeliet P. Placental growth factor (PlGF) and its receptor flt-1 (VEGFR-1). Ann N Y Acad Sci. (2002) 979(1):80–93. 10.1111/j.1749-6632.2002.tb04870.x 12543719

[37] Marco GSD Reuter S Hillebrand U Amler S König M Larger E The soluble VEGF receptor sFlt1 contributes to endothelial dysfunction in CKD. J Am Soc Nephrol. (2009) 20(10):2235–45. 10.1681/ASN.2009010061 19608702 PMC2754110

[38] Kishuku M Nishioka Y Abe S Kishi J Ogino H Aono Y Expression of soluble vascular endothelial growth factor receptor-1 in human monocyte-derived mature dendritic cells contributes to their antiangiogenic property. J Immunol. (2009) 183(12):8176–85. 10.4049/jimmunol.0803849 20007583

[39] Hong J Crawford K Cavanagh E Clifton V Kumar S. Prediction of preterm birth in women with fetal growth restriction—is the weekly change in sFlt-1/PlGF ratio or PlGF levels useful? Acta Obstet Gynecol Scand. (2024) 103(6):1112–1119. 10.1111/aogs.14831 38483020 PMC11103152

[40] Mijal RS Holzman CB Rana S Karumanchi SA Wang J Sikorskii A. Mid-pregnancy levels of angiogenic markers as indicators of pathways to preterm delivery. J Matern Fetal Neonatal Med. (2012) 25(7):1135–41. 10.3109/14767058.2011.625458 21939291 PMC4151045

[41] Chaparro A Zúñiga E Varas-Godoy M Albers D Ramírez V Hernández M Periodontitis and placental growth factor in oral fluids are early pregnancy predictors of gestational diabetes mellitus. J Periodontol. (2018) 89(9):1052–60. 10.1002/JPER.17-0497 29790168

[42] Chaparro A Gaedechens D Ramírez V Zuñiga E Kusanovic JP Inostroza C Placental biomarkers and angiogenic factors in oral fluids of patients with preeclampsia. Prenat Diagn. (2016) 36(5):476–82. 10.1002/pd.4811 26988336

[43] Yoo S-A Yoon H-J Kim H-S Chae C-B Falco SD Cho C-S Role of placenta growth factor and its receptor flt-1 in rheumatoid inflammation: a link between angiogenesis and inflammation. Arthritis Rheum. (2009) 60(2):345–54. 10.1002/art.24289 19180491

[44] Horton AL Boggess KA Moss KL Beck J Offenbacher S. Maternal periodontal disease and soluble fms-like tyrosine kinase-1 expression. J Periodontol. (2009) 80(9):1506–10. 10.1902/jop.2009.090189 19722802

[45] Vogt M Sallum AW Cecatti JG Morais SS. Periodontal disease and some adverse perinatal outcomes in a cohort of low risk pregnant women. Reprod Health. (2010) 7:29. 10.1186/1742-4755-7-29 21047427 PMC2987758

[46] Tellapragada C Eshwara VK Bhat P Acharya S Kamath A Bhat S Risk factors for preterm birth and low birth weight among pregnant Indian women: a hospital-based prospective study. J Prev Med Public Health. (2016) 49(3):165–75. 10.3961/jpmph.16.022 27255075 PMC4898897

[47] Agueda A Ramon JM Manau C Guerrero A Echeverria JJ. Periodontal disease as a risk factor for adverse pregnancy outcomes: a prospective cohort study. J Clin Periodontol. (2008) 35(1):16–22. 10.1111/j.1600-051X.2007.01166.x 18034850

[48] Offenbacher S Boggess KA Murtha AP Jared HL Lieff S McKaig RG Progressive periodontal disease and risk of very preterm delivery. Obstet Gynecol. (2006) 107(1):29–36. 10.1097/01.AOG.0000190212.87012.96. Erratum in: *Obstet Gynecol*. (2006) 107(5):1171. 16394036

[49] Rakoto-Alson S Tenenbaum H Davideau JL. Periodontal diseases, preterm births, and low birth weight: findings from a homogeneous cohort of women in Madagascar. J Periodontol. (2010) 81(2):205–13. 10.1902/jop.2009.090351 20151798

[50] Riché EL Boggess KA Lieff S Murtha AP Auten RL Beck JD Periodontal disease increases the risk of preterm delivery among preeclamptic women. Ann Periodontol. (2002) 7(1):95–101. 10.1902/annals.2002.7.1.95 16013222

[51] Al Habashneh R Khader YS Jabali OA Alchalabi H. Prediction of preterm and low birth weight delivery by maternal periodontal parameters: receiver operating characteristic (ROC) curve analysis. Matern Child Health J. (2013) 17(2):299–306. 10.1007/s10995-012-0974-2 22392602

[52] Wang YL Liou JD Pan WL. Association between maternal periodontal disease and preterm delivery and low birth weight. Taiwan J Obstet Gynecol. (2013) 52(1):71–6. 10.1016/j.tjog.2013.01.011 23548222

[53] Srinivas SK Sammel MD Stamilio DM Clothier B Jeffcoat MK Parry S Periodontal disease and adverse pregnancy outcomes: is there an association? Am J Obstet Gynecol. (2009) 200(5):497.e1–8. 10.1016/j.ajog.2009.03.003 19375568

